# Radiation-induced immunogenic modulation of tumor enhances antigen processing and calreticulin exposure, resulting in enhanced T-cell killing

**DOI:** 10.18632/oncotarget.1719

**Published:** 2013-12-31

**Authors:** Sofia R. Gameiro, Momodou L. Jammed, Max M. Wattenberg, Kwong Y. Tsang, Soldano Ferrone, James W. Hodge

**Affiliations:** ^1^ Laboratory of Tumor Immunology and Biology, Center for Cancer Research, National Cancer Institute, National Institutes of Health, Bethesda, Maryland, USA; ^2^ Department of Surgery, Massachusetts General Hospital, Boston, Massachusetts, USA

**Keywords:** radiation, immunogenic modulation, antigen-processing machinery, calreticulin, CTL, ER stress, PERK, TAP1, TAP2, calnexin, tapasin, LMP2, LMP7, LMP10

## Abstract

Radiation therapy (RT) is used for local tumor control through direct killing of tumor cells. Radiation-induced cell death can trigger tumor antigen-specific immune responses, but these are often noncurative. Radiation has been demonstrated to induce immunogenic modulation (IM) in various tumor types by altering the biology of surviving cells to render them more susceptible to T cell-mediated killing. Little is known about the mechanism(s) underlying IM elicited by sub-lethal radiation dosing. We have examined the molecular and immunogenic consequences of radiation exposure in breast, lung, and prostate human carcinoma cells. Radiation induced secretion of ATP and HMGB1 in both dying and surviving tumor cells. *In vitro* and *in vivo* tumor irradiation induced significant upregulation of multiple components of the antigen-processing machinery and calreticulin cell-surface expression. Augmented CTL lysis specific for several tumor-associated antigens was largely dictated by the presence of calreticulin on the surface of tumor cells and constituted an adaptive response to endoplasmic reticulum stress, mediated by activation of the unfolded protein response.

This study provides evidence that radiation induces a continuum of immunogenic alterations in tumor biology, from immunogenic modulation to immunogenic cell death. We also expand the concept of immunogenic modulation, where surviving tumor cells recovering from radiation-induced endoplasmic reticulum stress become more sensitive to CTL killing. These observations offer a rationale for the combined use of radiation with immunotherapy, including for patients failing RT alone.

## INTRODUCTION

Radiation therapy (RT) is standard of care for multiple malignancies, including carcinomas of the breast, lung, and prostate. Whether the intent is cure or palliation, the goal of RT is direct tumor-cell killing and/or modulation of tumor or stromal architecture. However, dose delivery constraints dictated by the need to limit damage to healthy tissues often result in dose heterogeneity in a given tumor mass, where not all tumor cells are exposed to a lethal dose of radiation.

Preclinical studies in various tumor models have shown that exposing tumor cells to lethal doses of radiation can elicit cell death while inducing strong antitumor immunity in a process initially described by Zitvogel and Kroemer *et al.* as immunogenic cell death (ICD) [1–3]. However, in a clinical setting, immune responses elicited by radiation alone rarely result in protective immunity, as locoregional relapse often occurs [4, 5]. Recent studies have demonstrated that RT has immunomodulatory consequences through direct action on surviving tumor cells and/or cells of the immune system [6–10]. We previously reported that radiation alters the biology of surviving tumor cells, rendering them more susceptible to T cell-mediated killing [6, 8]. We also demonstrated in both preclinical and clinical studies that radiation combined with vaccine elicits greater tumor antigen-specific CD8^+^ T-cell responses and/or reduction in tumor burden than either modality alone [10, 11]. Here, we examined radiation's ability to induce immunogenic modulation (IM) of breast cancer, non-small cell lung cancer (NSCLC), and prostate cancer cells, thus increasing their susceptibility to CD8^+^ CTL-mediated lysis. Importantly, we also examined the molecular mechanisms associated with IM of tumors. T cell-mediated killing relies on the recognition of specific CD8^+^-restricted epitopes associated with MHC class I molecules on the surface of tumor cells, which is dictated by the cooperative action of multiple elements of the antigen-processing machinery (APM). Mounting evidence suggests that APM defects in tumor cells have a detrimental effect on T-cell recognition [12–15]. Radiation has been shown to modulate the peptide repertoire, enhance MHC I expression, and increase the activity of TAP 1 [6, 8, 16]. We hypothesized that radiation could induce IM of tumor-cell phenotype and APM components, thereby enhancing productive interactions between CD8^+^ CTLs and tumor cells. Thus, we examined the effects of radiation on molecules that have been implicated in enhancing CTL-mediated tumor lysis, including calreticulin and APM components [17]. These studies are the first to report (a) an evaluation of cardinal signs of ICD and immunogenic modulation following radiation of breast, lung, and prostate carcinoma cell lines, (b) the use of radiation to functionally increase expression of APM components *in vitro* and *in vivo*, and (c) the functional role of calreticulin cell-surface expression and endoplasmic reticulum (ER) stress on IM and increased sensitivity to CTL killing of tumor cells that survive radiotherapy. These findings offer a rationale for the use of radiation in combination with immunotherapy, including for patients who have failed radiation therapy or have limited treatment options.

## RESULTS

### Radiation induces dose-dependent alterations in human carcinoma cells ranging from immunogenic modulation to cardinal signs of immunogenic cell death

High-dose radiation has been reported to induce ICD in preclinical models [1,2]. The cardinal signs of ICD are (a) calreticulin exposure on the surface of dying cells, (b) secretion of high-mobility group box 1 (HMGB1) protein, (c) release of ATP, and most importantly, (d) cell death. Each of these molecules stimulates dendritic cells to promote heightened immune responses [2, 18–20].

**Figure 1:**
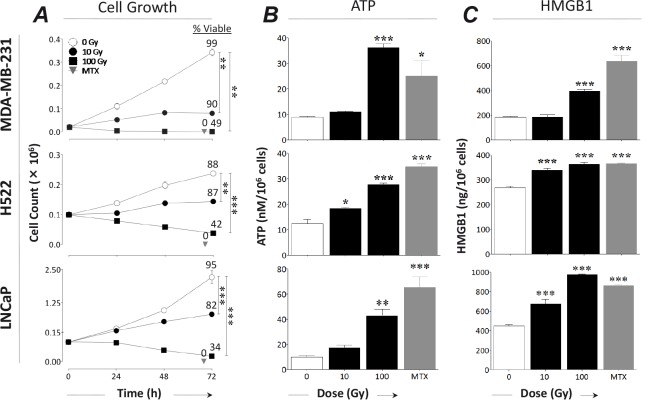
Radiation induces a continuum of dose-dependent cellular changes, ranging from immunogenic modulation to immunogenic cell death Human breast (MDA-MB-231), lung (H522), and prostate (LNCaP) carcinoma cells were exposed to 10 or 100 Gy (black circles, squares, and bars), or mock-irradiated with 0 Gy (open bars and circles). Mitoxantrone (MTX; 1 μM; gray bars and triangles) was used as a positive control for ICD. A, cell growth. Viability is indicated by percentage of 7-AAD- cells. B, ATP secretion. C, HMGB1 secretion. Results are presented as mean ± S.E.M. from 3 replicate wells. Asterisks denote statistical significance relative to mock-irradiated cells (*P < 0.05, **P < 0.01, ***P < 0.001). This experiment was repeated 3 times with similar results.

We first examined the *in vitro* effect of different doses of radiation on growth, viability, and cardinal signs of ICD in 3 human carcinoma cell lines: breast (MDA-MB-231), lung (H522), and prostate (LNCaP). Cells were mock-irradiated (0 Gy) or subjected to 10 or 100 Gy. Mitoxantrone was used as a positive control to induce ICD [20]. Exposure to 100 Gy significantly decreased growth and viability over 72 h in all cell lines relative to controls *(P* < 0.0001) (Fig. 1A). In each cell line, cells exposed to 100 Gy showed < 50% viability at 72 h post-irradiation and released significant amounts of ATP (Fig. 1B) and HMGB1 (Fig. 1C). In contrast, 10 Gy significantly reduced growth in all cell lines (*P* ≤ 0.005) without significantly reducing viability (Fig. 1A), yet also induced significant ATP release in lung cancer cells (Fig. 1B; *P* = 0.0002) and HMGB1 secretion in lung (*P* = 0.0003) and prostate (*P* = 0.0007) cancer cells (Fig. 1C). Mitoxantrone-treated cells were not viable 72 h post-treatment and released significant amounts of ATP (*P* ≤ 0.007) and HMGB1 (*P* ≤ 0.003) relative to controls. These data indicate that radiation induces dose-dependent immunogenic alterations in human carcinoma cells ranging from cardinal signs of ICD to immunogenic modulation.

### Sublethal irradiation of human carcinoma cells significantly increases sensitivity to antigen-specific CTL lysis

**Figure 2:**
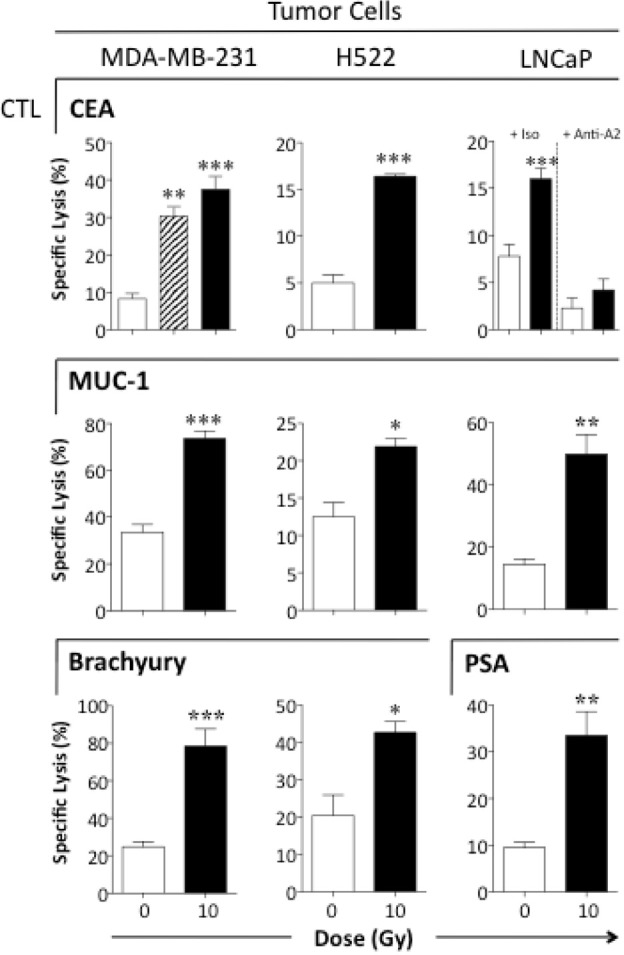
Tumor cells exposed to sublethal radiation demonstrate significantly increased sensitivity to antigen-specific CTL killing Human breast (MDA-MB-231), lung (H522), and prostate (LNCaP) carcinoma cells were mock-irradiated (0 Gy; open bars) or exposed to a single dose of 10 Gy (black bars). Indicated cells were exposed to fractionated radiation (2 Gy × 5; hatched bars). After 72 h, cells were used as targets in a CTL lysis assay using CEA-, MUC-1-, brachyury-, or PSA-specific CD8^+^ T cells as effector cells at an E:T ratio of 30:1. To verify CTL specificity, tumor cells were incubated with anti-HLA-A2 mAb or matched isotype control. Results are presented as mean ± S.E.M. from 3–6 replicate wells. Asterisks denote statistical significance relative to controls (*P < 0.05, **P < 0.01, ***p < 0.001). This experiment was repeated 2–5 times with similar results.

Exposing human HNSCC, colon, and prostate carcinoma cells to sublethal irradiation has previously been shown to increase the sensitivity of tumor cells to CTL lysis [6, 8, 21]. We sought to confirm and extend these findings to breast (MDA-MB-231), lung (H522), and prostate (LNCaP) carcinoma cells. MDA-MB-231 cells were mock-irradiated (0 Gy) or given 10 Gy as a single dose or 2 Gy/day for 5 days. As shown in Figure 2, 72 h after exposure to 10 Gy in either a single or fractionated dose, breast carcinoma cells were significantly more sensitive to CEA-specific T cell-mediated lysis (*P* = 0.0006). Since results were similar with single and fractionated dosing, we chose to use a single 10-Gy dose in subsequent experiments. Exposing breast carcinoma cells to 10 Gy also increased their sensitivity to MUC-1- (*P* = 0.001) and brachyury-specific T-cell lysis (*P* = 0.005), and significantly increased the sensitivity of lung and prostate carcinoma cells to CTLs specific for CEA (*P* = 0.0002, *P* < 0.0001, respectively), MUC-1 (*P* = 0.013, *P* = 0.006, respectively), and brachyury (*P* = 0.0002, *P* < 0.0001, respectively). Irradiated prostate carcinoma cells were also more sensitive to PSA-specific lysis relative to controls (*P* = 0.01). CTL killing was MHC I-restricted as determined by anti-HLA-A2 blocking antibody (*P* < 0.0001) (Fig. 2).

These data indicate that exposure to sublethal doses of radiation enhances antigen-specific CTL-mediated killing across multiple carcinoma types, and that this effect can be extended to a variety of tumor-associated antigens (TAAs).

### Sublethal radiation causes immunogenic modulation in carcinoma cells, including increased APM component expression and cell-surface expression of calreticulin

**Figure 3:**
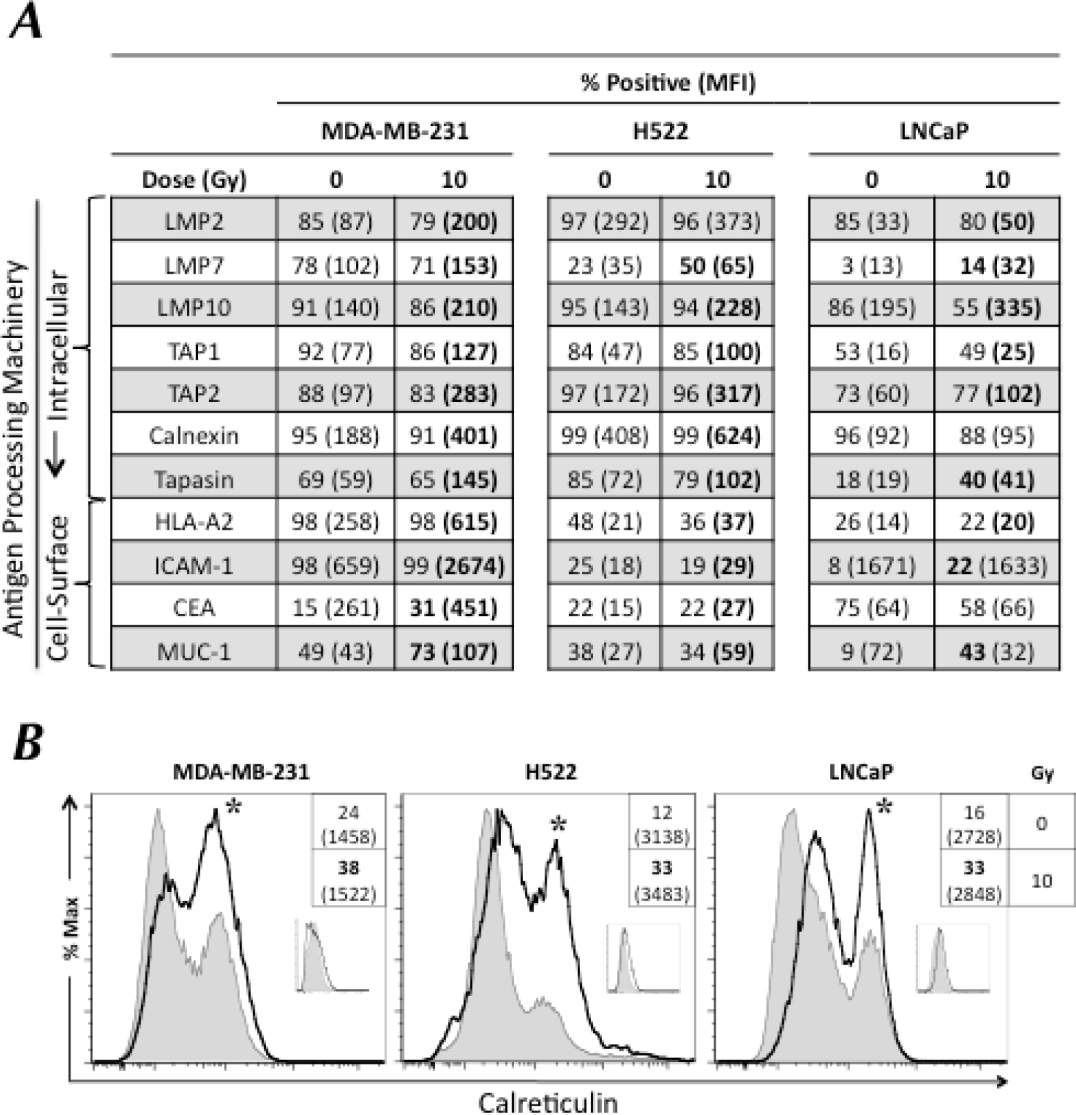
Tumor cells exposed to sublethal radiation undergo immunogenic modulation and demonstrate increased cell-surface expression of calreticulin Human breast (MDA-MB-231), lung (H522), and prostate (LNCaP) carcinoma cells were mock-irradiated (0 Gy) or exposed to a single dose of 10 Gy radiation. After 72 h, cells were analyzed by flow cytometry. A, intracellular expression of indicated APM components and cell-surface expression of HLA-A2, ICAM-1, CEA, and MUC-1. B, cell-surface expression of calreticulin in tumor cells exposed to 10 Gy (open histograms) or mock-irradiated (0 Gy; shaded histograms). Insets depict binding of isotype-matched control antibodies. Numbers indicate percentage of positive cells. Numbers in parentheses denote MFI. Bold denotes marked upregulation (≥ 30% increase in percent of cells or mean fluorescence intensity (MFI) not observed in isotype control vs. untreated cells). Asterisks denote statistical significance relative to mock-irradiated cells. This experiment was repeated 3 times with similar results.

Tumor cells' increased sensitivity to CTL killing suggests enhanced T-cell recognition of specific HLA I/CD8^+^-restricted epitope complexes on the surface of tumor cells. The presence and repertoire of MHC I/epitope complexes on the surface of tumor cells is dictated by the cooperative interactions of multiple APM components. Hypothesizing that radiation could modulate the expression of APM components, we exposed breast (MDA-MB-231), lung (H522), and prostate (LNCaP) carcinoma cells to 0 or 10 Gy. At 72 h post-irradiation, we examined cells by flow cytometry for intracellular expression of 7 APM components (Fig. 3A). In each tumor cell line, exposure to radiation significantly increased the expression of ≥ 6 APM components by ≥ 30%, namely the immunoproteosome subunits LMP2, LMP7, and LMP10; the peptide transporters TAP1 and TAP2; and the chaperones calnexin (2/3 cell lines) and tapasin (3/3 cell lines). These data indicate that sublethal radiation induces significant and widespread upregulation of most APM components across multiple tumor types, which is likely to result in increased T-cell recognition and lysis of irradiated tumor targets.

Because several proteins present on the surface of tumor cells have previously been demonstrated to be critical for interactions with CD8^+^ T cells [6], we also examined the effect of sublethal radiation on phenotypic modulation of breast, lung, and prostate carcinoma targets. We exposed cells to 0 or 10 Gy and monitored cell-surface expression of MHC I, ICAM-1, CEA, MUC-1, and calreticulin by flow cytometry 72 h post-treatment. As shown in Figure 3A, exposure to radiation induced significant cell-surface expression of MHC I (3/3 cell lines), ICAM-1 (3/3), CEA (2/3), and MUC-1 (3/3), regardless of carcinoma type. We also observed a significant translocation of the ER chaperone calreticulin to the cell surface 72 h after exposure to sublethal radiation in 3/3 cell lines (Fig. 3B). Taken together, these data indicate that exposure of human carcinoma cells to sublethal radiation induces IM across disparate tumor types, as evidenced by increased expression of multiple APM components and translocation of calreticulin to the cell surface of tumor targets in the absence of cell death.

### Exposure to radiation *in vivo* increases expression of APM components and promotes cell-surface expression of calreticulin in carcinoma cells

**Figure 4:**
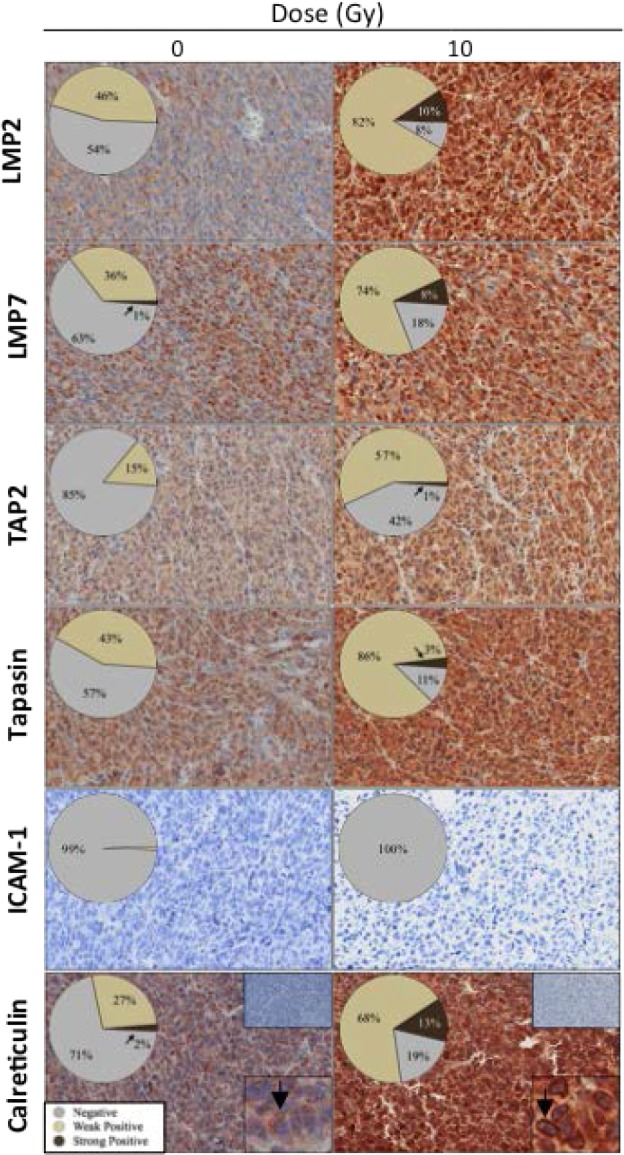
Exposure of tumors to radiation *in vivo* increases expression of APM components and promotes cell-surface expression of calreticulin Nude mice bearing LNCaP prostate xenografts were exposed to a single dose of 10 Gy or left untreated (0 Gy). A, after 3 days, tumors were surgically removed, and evaluated by immunohistochemistry (40 ×) for expression of ICAM-1 and the APM components LMP2, LMP7, TAP2, tapasin, and calreticulin. Numbers indicate percentage of cells with negative expression, weak/medium positive, or strong positive, as determined by pixel analysis (*n* = 2 mice/treatment group). Calreticulin upper insets: isotype control. Calreticulin lower insets: arrow indicates calreticulin membrane staining.

To confirm that *in vivo* exposure to sublethal radiation modulates APM elements and calreticulin translocation to the surface of carcinoma cells, nude mice (*n* = 2/group) were implanted with LNCaP tumors and exposed to 0 or 10 Gy. Immunohistochemistry analysis of prostate carcinomas 72 h post-irradiation showed increased expression of the immunoproteosome components LMP2 and LMP7, the peptide transporter TAP2, and the chaperones tapasin and calreticulin (Fig. 4). However, tumor irradiation did not modulate ICAM-1 expression. Pixel analysis of one representative tumor indicated that < 50% of tumor cells in non-irradiated carcinomas were positive for LMP2, but none exhibited strong protein levels. However, post-irradiation, the population of tumor cells expressing LMP2 increased 2-fold, with 10% showing strong LMP2 expression. Similar increases in LMP2 expression were observed in all tumors exposed to radiation. Radiation also induced a marked increase in expression of TAP2 (3.9-fold) and tapasin (2.1-fold average) relative to untreated controls. Calreticulin is heterogeneously expressed in untreated LNCaP tumors. As seen in Figure 4, calreticulin was absent in > 70% of tumor cells, with only 2% expressing strong protein levels. Further, calreticulin localization was diffuse (inset panel). However, after exposure to radiation, positive calreticulin expression increased > 1.9-fold on average, a significant upregulation relative to controls. Radiation also caused a 6.5-fold increase in tumor cells with strong calreticulin expression relative to non-irradiated controls. Moreover, calreticulin expression in carcinoma cells exposed to radiation was associated with the cell membrane (inset, black arrows). In non-irradiated tumors, calreticulin membrane staining was 34.7% ± 20.4% vs. tumors exposed to radiation, where calreticulin membrane staining increased to 78.7% ± 13.1%. These data confirm that radiation is an immunogenic modulator *in vivo*, as evidenced by widespread upregulation of APM components and increased translocation of calreticulin to the surface of carcinoma cells.

In a separate experiment where LNCaP xenografts (*n* = 5/group) were examined 72 h after exposure to 10 Gy as a single dose or in daily fractions of 2 Gy, no significant differences in LMP2, LMP7, TAP2, or tapasin expression were observed between both treatments, suggesting that radiation therapy modulates the expression of APM components regardless of dosing regimen (data not shown).

### Exposure of tumor cells to sublethal radiation increases expression of surface calreticulin, which leads to increased sensitivity to CTL-mediated lysis

**Figure 5:**
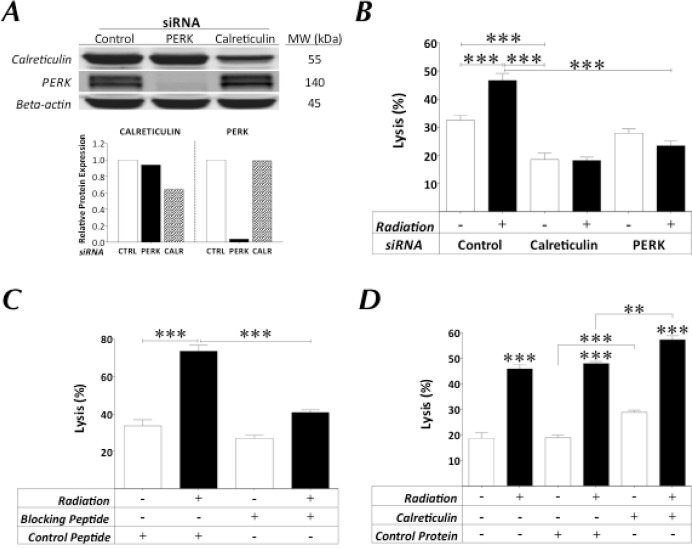
Expression of surface calreticulin in tumor cells exposed to sublethal radiation dictates increased sensitivity to CTL-mediated lysis A, protein expression by Western blotting of MDA-MB-231 tumor cells exposed to control, calreticulin. or PERK siRNA knockdown. Lower panel denotes protein expression of calreticulin or PERK relative to beta-actin. B, MDA-MB-231 carcinoma cells with control, calreticulin, or PERK knockdown were mock-irradiated (0 Gy; open bars) or exposed to a single dose of 10 Gy (black bars). After 48 h, cells were used as targets in a CEA-specific CTL-lysis assay. For selected experiments, MDA-MB-231 tumor cells were exposed to 0 or 10 Gy 72 h prior to being used as targets in a CEA-specific CTL-lysis assay in the presence of (C) calreticulin-blocking or LCMV control peptides or (D) human calreticulin or serum albumin proteins. CD8^+^ T cells were used as effector cells at an E:T ratio of 30:1. Results are presented as mean ± S.E.M. from 3–6 replicate wells. Asterisks denote statistical significance relative to controls (**P < 0.01, ***p < 0.001). Data represent 3 independent experiments.

Calreticulin is a critical component of antigen processing and loading into MHC I [22, 23]. Based on its upregulation and, more importantly, its translocation to the cell surface across disparate tumor types (Figs. 3B and 4) in response to sublethal radiation, we hypothesized that surface calreticulin plays an additional role in IM as it pertains to increased CTL-mediated lysis. We analyzed the role of surface calreticulin in IM by: (a) siRNA knockdown of the serine/threonine kinase PERK, which mediates translocation of calreticulin to the cell surface, where knockdown of calreticulin was used as a positive control [3], (b) use of a calreticulin-blocking peptide [24], and (c) addition of exogenous calreticulin protein. We expected that knockdown of calreticulin would prevent normal APM functions and thus impair T-cell recognition of tumor [22]; that silencing of PERK would maintain APM functions but prevent calreticulin translocation [3, 17]; and that peptide blocking would prevent surface calreticulin/T-cell interactions, whereas the presence of exogenous calreticulin would promote those interactions. Following RNA interference in MDA-MB-231 cells, total expression of calreticulin and PERK decreased by 36% and 96%, respectively (Fig. 5A). In both cases, the expression of calreticulin on the cell surface was abrogated without decreasing MHC I or ICAM-1 expression (Supplemental Fig. 1). As before, MDA-MB-231 cells were lysed by CEA-specific T cells to a significantly greater extent after exposure to 10 Gy radiation (Fig. 5B; *P* < 0.0001). However, this increased killing was abrogated in carcinoma cells with reduced expression of calreticulin or PERK (*P* < 0.0001). Similar results were observed with LNCaP cells (data not shown). In addition, MDA-MB-231 cells co-incubated with control peptide were killed to a significantly greater level after exposure to radiation (Fig. 5C; *P* < 0.0001). However, in the presence of calreticulin-blocking peptide, the radiation-induced increase in CTL lysis of MDA-MB-231 carcinoma cells was significantly reduced (Fig. 5C; *P* < 0.0001). As a control, incubation of the CEA-specific CTL used in these assays with calreticulin-blocking peptide did not affect T-cell viability (data not shown). Further, MDA-MB-231 cells co-incubated in the absence or presence of control protein were equally lysed, and to a significantly higher level after exposure to radiation (Fig. 5D; *P* < 0.0001). However, in the presence of exogenous calreticulin, the level of CTL lysis of MDA-MB-231 carcinoma cells exposed to radiation increased further relative to irradiated targets plus control protein (*P* < 0.0001). Exogenous calreticulin also augmented CTL-mediated lysis of non-irradiated carcinoma targets relative to targets plus control protein (*P* < 0.0001). Collectively, these data indicate that increased sensitivity of carcinoma cells to T cell-mediated lysis is dependent upon the presence of calreticulin on the cell surface.

### Radiation-induced immunogenic modulation of carcinoma cells is mediated by ER stress

**Figure 6:**
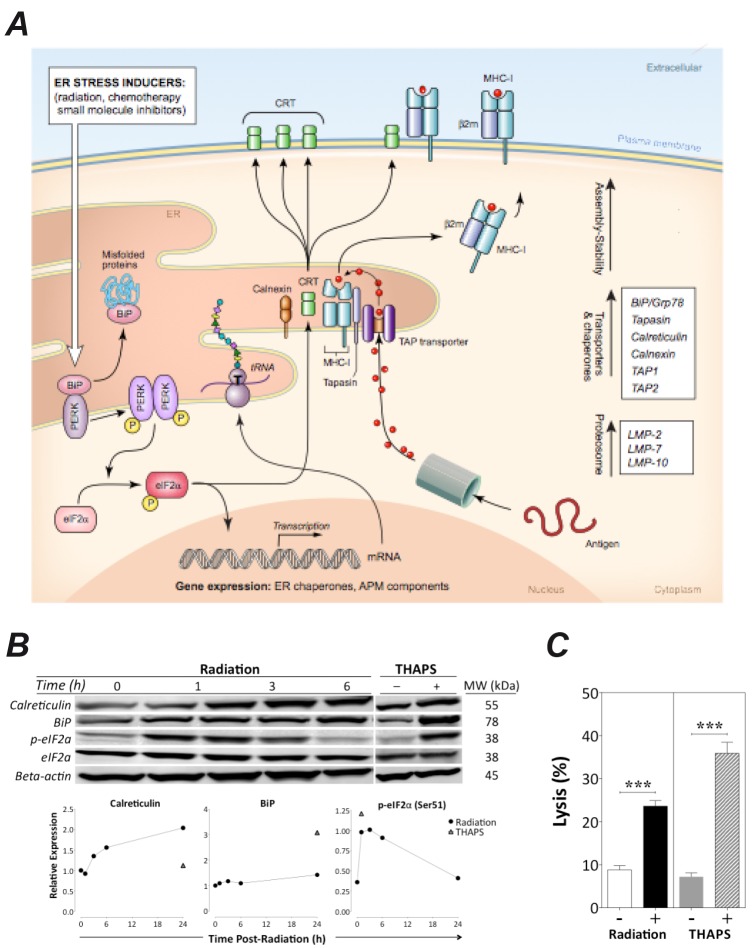
Radiation-induced immunogenic modulation of MDA-MB-231 carcinoma cells is mediated by ER stress A, schematic model of cell response to radiation-induced ER stress. B, at indicated times after exposure of MDA-MB-231 cells to 0 or 10 Gy, total cell lysates were examined by Western blotting to determine expression of calreticulin, BiP, total eIF2a, and eIF2a-pSer51. Exposure to thapsigargin was used as positive control for ER stress. Beta-actin was used as an internal control for total protein levels. Lower panels denote protein expression relative to beta-actin. This experiment was repeated twice with similar results. C, MDA-MB-231 cells were mock-irradiated (0 Gy; open bar), exposed to 10 Gy (black bar), or treated with thapsigargin (hatched bar) or DMSO as control (grey bar). After 48 h, cells were used as targets in a CTL-lysis assay where effector CEA-specific CD8^+^ T cells were used at an E:T ratio of 30:1. Results are presented as mean ± S.E.M. from 6 replicate wells. Asterisks denote statistical significance relative to controls (***P < 0.001). This experiment was performed 3 times attaining similar results.

Calreticulin exposure and induction of ER molecular chaperones can occur under certain conditions of ER stress [25]. ER stress triggers the unfolded protein response (UPR), an evolutionarily conserved quality control mechanism, which attempts to restore ER homeostasis through a cascade of cellular events, including transient arrest of protein translation and upregulation of ER protein chaperones. We hypothesized that radiation induces ER stress on tumor cells, which then triggers an adaptive response through the UPR, resulting in immunogenic modulation and increased sensitivity to CTL killing (Fig. 6A). We examined the effect of radiation on expression of various proteins induced by the UPR in response to ER stress. Induction of calreticulin and BiP (or Grp78), 2 abundant ER chaperone proteins with important roles in the maintenance of ER homeostasis, is a symptom of ER stress [15, 23, 26]. Phosphorylation of eIF2a occurs in response to ER stress through the PERK-mediated branch of the UPR [26].

We exposed MDA-MB-231 cells to 0 or 10 Gy and examined levels of calreticulin, BiP, total eIF2a, and phosphorylated eIF2a at the indicated time points. Tumor cells exposed to the ER stress inducer thapsigargin were used as positive controls. Radiation increased the expression of calreticulin, BiP, and phosphorylated eIF2a in a time-dependent manner, similar to that observed after thapsigargin treatment, indicating that radiation induces ER stress and that in response to it, cells upregulate APM components (Fig. 6B and Supplemental Fig. 2). Identical results were obtained with LNCaP cells (data not shown). Thapsigargin treatment of MDA-MB-231 cells significantly increased cell-surface expression of HLA-A2, ICAM-1, and MUC-1, without modulating CEA protein levels (Supplemental Table 1). Similarly to radiation, thapsigargin-induced ER stress significantly increased the intracellular expression of multiple APM components, including LMP2, LMP7, TAP1, TAP2, and the protein chaperones calnexin, tapasin, and calreticulin (Supplemental Table 1). However, and in contrast to radiation, it did not promote calreticulin translocation to the surface of MDA-MB-231 cells (not shown), in accordance with other reports [3]. These data indicate that the survival response orchestrated by human carcinoma cells in reaction to sublethal radiation includes the PERK-mediated branch of the UPR. To examine the functional consequence of radiation-induced ER stress/UPR, we treated MDA-MB-231 cells as previously described and used them 48 h later as targets in a CEA-specific CTL lysis assay. As before, carcinoma-cell lysis increased significantly after exposure to radiation (Fig. 6C). Importantly, lysis of carcinoma cells significantly increased after exposure to thapsigargin (*P* < 0.0001), which had no effect on cellular viability, as determined by 7-AAD exclusion (data not shown). Collectively, these data suggest that the increased sensitivity of human carcinoma cells to CTL-mediated lysis after exposure to radiation is a result of a cellular survival response to ER stress mediated through the UPR.

## DISCUSSION

Radiation has been shown to induce IM in a wide variety of carcinoma types by altering the biology of surviving tumor cells to render them more susceptible to T cell-mediated killing [6, 8]. Radiation-induced tumor-cell death can occur through multiple mechanisms [27]. Studies in preclinical models have shown that radiation-induced tumor-cell death is immunogenic and thus elicits strong antitumor immunity, a process described by Zitvogel and Kroemer *et al*. as immunogenic cell death (ICD) [1–3]. However, immune responses observed in patients treated with radiation alone are often weak and do not result in protective immunity [4, 11].

Here we report that radiation induces cardinal signs of ICD (cell death and release of ATP and HMGB1) in multiple carcinoma types (Fig. 1). The highest dose used here (100 Gy) is above the clinical range and was selected to ensure cell death in all cell lines examined; however, the ATP and HMGB1 secretion we observed at a sublethal (10 Gy) dose suggests that ICD may be achieved clinically [4, 27]. We made similar observations in experiments with breast (MCF-7), lung (NCI-H1703), and prostate (DU-145 and PC-3) carcinoma cell lines (data not shown). These findings support the premise that therapeutic cancer vaccines can exploit the immunostimulatory environment created by RT to achieve robust and effective antitumor immune responses in multiple carcinomas [9, 10, 28, 29].

This concept is further supported by our findings that tumor cells of distinct origin and genotype that survive radiation exposure are significantly more sensitive to CTL lysis (Fig. 2). Interestingly, the cells' sensitivity to CTL lysis was enhanced regardless of their p53 or K-Ras mutation status or triple-negative phenotype. Mutations in p53 occur in ~ 50% of cancers, including ~ 80% of triple-negative breast cancers, and render tumors resistant to RT, whereas the frequent presence of K-Ras mutations in NSCLC or triple-negative breast cancers severely limits treatment options [27, 30–32]. This effect can be extended to a variety of TAAs, expanding prior observations in carcinomas of the head and neck, colon, and prostate [6, 8, 21].

Distinct gene signatures have been reported in human prostate and breast carcinoma cells exposed *in vitro* and *in vivo* to single (10 Gy) or fractionated (2 Gy × 5) doses of radiation [32, 33]. Despite different molecular profles, our data (Fig. 2) indicate that radiation-induced tumor sensitivity to CTL lysis is equally augmented with single or fractionated doses of radiation, suggesting that either regimen could elicit effective antitumor immune responses when combined with a therapeutic cancer vaccine. Further studies are needed to determine which RT regimens and doses best synergize with immunotherapy.

T cell-mediated attack is induced when CD8^+^ T cells recognize specific MHC I/epitope complexes on the surface of tumor cells, the presence of which relies on the proper function of multiple APM components (Fig. 6A). Defective expression and/or function of APM components results in poor CD8^+^ T-cell recognition [12–14]. APM defects have been identified in established human cancer cell lines and patient lesions, and have been correlated with poor clinical outcome across multiple tumor types [34, 35]. Here we demonstrate that exposure of human breast, lung, and prostate carcinoma cells to sublethal radiation induces a significant increase in protein expression of multiple APM components, including that of immunoproteosome subunits, peptide transporters, and protein chaperones, both *in vitro* (Fig. 3) and *in vivo* (Fig. 4). Similar results were observed *in vitro* with additional breast (MCF-7) and lung (NCI-H1703) carcinoma cells (data not shown). Although ICAM-1 expression was modulated following radiation exposure *in vitro* (Fig. 3), it was not upregulated after radiation exposure *in vivo* (Fig. 4), which suggests a role for the tumor microenvironment in ICAM-1 regulation, in agreement with other reports [36]. Further, no significant differences in APM modulation *in vivo* were observed between fractionated (2 Gy × 5) and single dose (10 Gy × 1) radiation. These results extend the findings of previous reports showing that radiation increases MHC I expression and TAP1 activity and alters the peptide repertoire presented by MHC I molecules [6, 16]. Based on the findings reported here (Figs. 1, 2, 3, and 4), it is reasonable to anticipate that tumor cells not eradicated by RT may undergo IM, thus becoming more susceptible to killing by vaccine-induced CTLs. This is supported by previous [7, 8] and current findings (Fig. 3) demonstrating increased expression of TAAs and other proteins contributing to efficient CTL lysis, such as MHC I and ICAM-1, following exposure of tumor cells to sublethal radiation. These findings also corroborate both preclinical and clinical studies demonstrating that radiation combined with vaccine elicits greater anti-tumor T-cell responses and/or reduction in tumor burden than either modality alone [11].

Calreticulin is a critical regulator of calcium homeostasis and a chaperone that assists in the folding of newly synthesized glycoproteins. Calreticulin is also an important component of the peptide-loading complex, where it plays a key role in assuring the export of properly loaded MHC I molecules [15]. When calreticulin is deficient, the majority of MHC I molecules are loaded with suboptimal peptides [15, 37]. Importantly, calreticulin maintains the low peptide threshold required for efficient antigen presentation [37]. Emerging data describe the role of calreticulin expressed on the surface of cells undergoing ICD, where it acts as a phagocytic signal for dendritic cells [38, 39].

Given that calreticulin expression was observed in cells treated *in vitro* and *in vivo* with sublethal doses of radiation (Figs. 3 and 4), and that calreticulin is critical for the enrichment of endogenous peptides in the ER and thus for efficient antigen presentation [37], we next investigated the role of this chaperone in IM. The presence of calreticulin on the tumor-cell surface requires PERK-mediated phosphorylation of the translation initiation factor eIF2a [40, 41]. Knockdown of PERK abrogated the translocation of calreticulin to the cell surface (Supplemental Fig. 1) and significantly reduced irradiated tumor cells' sensitivity to CTL lysis (Fig. 5B). As expected, abrogation of cell-surface expression (Supplemental Fig. 1) and decrease in total protein levels following siRNA knockdown of calreticulin (Fig. 5A) significantly inhibited CTL lysis of tumor cells exposed to sublethal radiation. These data concur with those of Garg *et al.*, who reported that PERK silencing abrogated the ability of calreticulin to translocate to the cell surface of murine tumor cells undergoing ICD [42]. Our data (Fig. 5C) suggest that the increased CTL lysis of tumor targets exposed to radiation is a result of the direct interaction of exposed calreticulin with CTLs, as the augmented CTL killing was abrogated in the presence of a calreticulin blocking peptide (Fig. 5C). Moreover, CTL lysis increased when exogenous calreticulin was added to non-irradiated tumor cells, and increased even further when exogenous calreticulin was added to irradiated tumor cells (Fig. 5D). These findings confirm and extend previous observations that while docetaxel failed to induce ICD in various human carcinoma cell lines, it did promote calreticulin exposure in cells treated with sublethal doses, resulting in increased CTL lysis. This contrasts with the role of calreticulin in ICD, where calreticulin exposure is not sufficient to elicit antitumor responses [41]. The specific molecules involved in calreticulin/T-cell interaction have not been definitively identified, but there are several potential candidates, including scavenger receptor A, CD69, low-density lipoprotein receptor-related protein 1 (LRP1, CD91), and perforin [23, 38, 39, 43].

Conditions of cellular stress, such as protein misfolding, that interfere with ER homeostasis are collectively referred to as ER stress. Mounting evidence suggests that calreticulin exposure in tumor cells can occur under multiple circumstances as a result of ER stress [1, 23–25]. Exposure of calreticulin has been observed in a variety of primary human cancer cells, in tumor cells subjected to mild disruption of calcium homeostasis, and in pre-apoptotic cancer cells undergoing ICD [1, 24, 25]. ER stress induces the evolutionarily conserved quality control mechanism known as UPR, which attempts to restore ER homeostasis through a cascade of molecular events, including transient arrest of protein translation and upregulation of ER protein chaperones. If this restoration fails, cells die. Here (Fig. 6B) we report that radiation induces ER stress in human carcinoma cells, triggering a survival response, which encompasses the PERK branch of the UPR, resulting in increased expression of multiple APM components and other immune-relevant proteins, consistent with observations with the ER-stress inducer thapsigargin (Figs. 3–4, Supplemental Table 1). Further, our observations (Figs. 5–6) also strongly suggest that IM and the consequent increased tumor sensitivity to CTL lysis are a result of this survival response orchestrated in reaction to radiation-induced ER stress. However, other mechanisms and/or branches of the UPR other than PERK may be induced by radiation to promote the observed effects, including the calreticulin role in CTL lysis.

This study provides evidence of a novel mechanism whereby RT induces a continuum of immunogenic alterations in tumor biology, ranging from IM to ICD. We expand the concept of IM to include tumor cells recovering from radiation-induced stress subsequently becoming more sensitive to CTL killing. These observations provide a rationale for the use of radiation in combination with immunotherapy, including for patients who have failed RT or who have limited treatment options.

## MATERIALS AND METHODS

### Tumor-cell lines

Cells of human breast carcinoma [MDA-MB-231 (ATCC® HTB-26 ™)], lung carcinoma [NCI-H522 (ATCC® CRL-5810™)], and prostate carcinoma [LNCaP clone FGC (ATCC® CRL-1740™)] were obtained from American Type Culture Collection (ATCC, Manassas, VA) and cultured in medium designated by the provider for propagation and maintenance.

### Tumor irradiation

Adherent tumor cells in log-growth phase were placed on ice and mock-irradiated or irradiated by a ^137^Cs source (Gammacell-1000, AECL/Nordion; Kanata, Ontario, Canada) at a dose rate of 5.56 Gy/min. Cells were then washed in fresh medium and incubated at 37°C with 5% CO_2_.

### Analysis of cell growth and cardinal signs of immunogenic cell death

Tumor cells exposed to 0, 10, or 100 Gy were incubated at 37°C with 5% CO_2_ for 3 days. Cells were harvested daily and viable cells were counted by trypan blue exclusion using a Cellometer Auto T4 automated cell counter (Nexcelom Bioscience, Lawrence, MA). Cellular viability was confirmed by 7-AAD (BD Biosciences, San Diego, CA) exclusion. Supernatants were analyzed for high-mobility group box 1 (HMGB1) and ATP contents 72 h after irradiation, as previously described [17], Mitoxantrone (1 μM, NDC 61703-343-65; Hospira, Lake Forest, IL) was used as a positive control for cell death and secretion of HMGB1 and ATP.

### Proteins, peptides, and chemicals

Human recombinant calreticulin was obtained from Enzo Life Sciences (Farmingdale, NY), human serum albumin and thapsigargin from Sigma (St. Louis, MO), and calreticulin blocking peptide from MBL International (Woburn, MA). The lymphocytic choriomeningitis virus peptide LCMV NP_118–132_ [RPQASGVYMGNLTAQ] was obtained from CPC Scientific (Sunnyvale, CA).

### CD8^+^ cytotoxic T-cell lines

CEA-specific CTLs recognize the CEA peptide epitope YLSGANLNL (CAP-1) [44, 45]. Prostate-specific antigen (PSA)-specific CTLs recognize the PSA peptide epitope VLSNDVCAQV [46]. The MUC-1-specific CD8^+^ CTL line, designated MUC-1 CTL, recognizes the MUC-1 peptide epitope ALWGQDVTSV [46]. Brachyury-specific CTLs recognize the brachyury peptide epitope WLLPGTSTL (T-p2) [47]. All T-cell lines were HLA-A2-restricted.

### Cytotoxicity assays

CTLs were used as previously described [17]. Tumor cells were mock irradiated (0 Gy) or exposed to 10 Gy in a single dose or in daily fractions of 2 Gy for 5 days. At 72 h post-irradiation, adherent cells were used as targets in a standard cytotoxicity assay using ^111^In [6, 21]. For indicated experiments, carcinoma cells were used as CTL targets 48 h post-exposure to 0 Gy, 10 Gy, thapsigargin (0.25 μM/1 h) or DMSO control. For indicated experiments, the CTL assay was performed in the presence of anti-HLA-A2 mAb (20 mg/mL; AbD Serotec, Raleigh, NC) or isotype control mAb (IgG2b, 20 mg/mL; AbD Serotec), exogenous calreticulin (9 nM, MW = 55 kDa) or control human serum albumin (MW = 65 kDa), calreticulin blocking peptide (1.7 μM, 1.43 kDa) or control LCMV_118–132_ peptide (MW = 1.59 kDa).

### Flow cytometry analysis

Cell-surface and intracytoplasmic staining was performed as previously described [48]. For indicated experiments, carcinoma cells were examined 48 h or 72 h post-exposure to 0 Gy, 10 Gy, thapsigargin (0.2 μM/1 h) or DMSO control. Surface staining of tumor cells was performed using the primary labeled monoclonal antibodies HLA-A2-FITC, ICAM-1 (CD54)-PE, CEA (CD66)-FITC, MUC-1 (CD227)-FITC, and the appropriate isotype-matched controls (BD Biosciences). Anti-calreticulin-PE and matched isotype control antibody were obtained from R&D Systems (Minneapolis, MN). For intracellular analysis of APM components, mouse IgG1 (MK2-23) isotype control, LMP2 (SY-1)-, LMP7 (HB2)-, LMP10 (TO-7)-, TAP-1 (NOB1)-, TAP-2 (NOB2)-, calnexin (TO-5)-, and tapasin (TO-3)-specific monoclonal antibodies were developed and characterized as described [49–51]. Cellular fluorescence of 3 × 10^4^ cells was examined on a FACSCalibur flow cytometer using CellQuest software (BD Biosciences). Proteins were scored as markedly upregulated if detection levels and/or mean fluorescence intensity (MFI) increased > 30% following treatment and were not observed in control cells vs. isotype controls.

### Calreticulin and PERK siRNA knockdown and Western blots

Silencer® siRNA and negative control siRNA were used to silence calreticulin and PERK in tumor cells, according to the manufacturer's instructions (Life Technologies, Grand Island, NY). For selected experiments, cells were irradiated with 0 or 10 Gy 24 h after being exposed to siRNA and used as CTL targets 48 h later. For protein blotting, tumor cells were lysed in RIPA buffer modified with 1 mM PMSF (Cell Signaling Technology, Beverly, MA) 48 h after gene knockdown. Proteins (20–40 μg) were resolved using 4%–12% Trisglycine SDS-PAGE (Life Technologies) then transferred to nitrocellulose membranes. Primary antibodies specific for PERK, calreticulin, BiP, eIF2a, phospho-eIF2a (Ser51), and beta-actin were acquired from Cell Signaling Technology. Blots were incubated with anti-rabbit IRDye secondary antibodies (LI-COR Biotechnology, Lincoln, NE), and detection was performed with the Odyssey Infrared Imaging System (LI-COR Biotechnology). Band density was quantified using ImageJ software (NIH, Bethesda, MD), and all protein levels were normalized to beta-actin. Phospho-eIF2a (Ser51) protein levels were further normalized to total eIF2a levels.

### *In-vivo* studies

Briefly, LNCaP tumor cells were implanted into nude mice (nu/nu) (Charles River, Wilmington, MA). When tumors reached a volume of 1000 mm^3^ they were exposed to 0, 10 Gy, or 2 Gy/day for 5 days. At 72 h postirradiation, tumors were surgically removed and paraffin sections were prepared. Tissues were then analyzed by immunohistochemistry for protein expression of LMP2, LMP7, TAP2, tapasin, ICAM-1, and calreticulin, then rinsed and counterstained with hematoxylin. Slides were digitally scanned by an Aperio ScanScope CS scanning system and, with the exclusion of necrotic regions, analyzed by Aperio Image Scope Viewer software (Aperio Technologies Inc., Vista, CA). Positive tumor regions were measured using the Positive Pixel Count v9 algorithm, as previously described [17]. All antibodies were purchased commercially and validated using adequate positive controls. Negative controls included omission of primary antibody with PBS and matched rabbit isotype antibody.

### Statistical analysis

Significant differences between multiple treatment groups were determined by 1-way ANOVA with Tukey's comparison, based on a confidence interval of 95% using Prism 5.0d software (GraphPad Software Inc., La Jolla, CA). Alternatively, statistical differences between 2 treatments were analyzed by unpaired Student's t test with a 2-tailed distribution, unless reported otherwise, and reported as P values. Significant differences in the distribution of flow cytometry analysis data were determined by the Kolmogorov-Smirnov test using CellQuest software (BD Biosciences).

## Supplementary Figures and Table




