# Supplementary material for: Radiation-induced immunogenic modulation of tumor enhances antigen processing and calreticulin exposure, resulting in enhanced T-cell killing
Source: Oncotarget. 2013 Dec 31;5(2):403–16. doi: 10.18632/oncotarget.1719 (PMC3964216; doi:10.18632/oncotarget.1719)
Supplement: Supplementary file 1 [file oncotarget-05-403-s001.pdf]

Radiation-induced immunogenic modulation of tumor enhances antigen processing and calreticulin exposure, resulting in enhanced T-cell killing - Gameiro et al

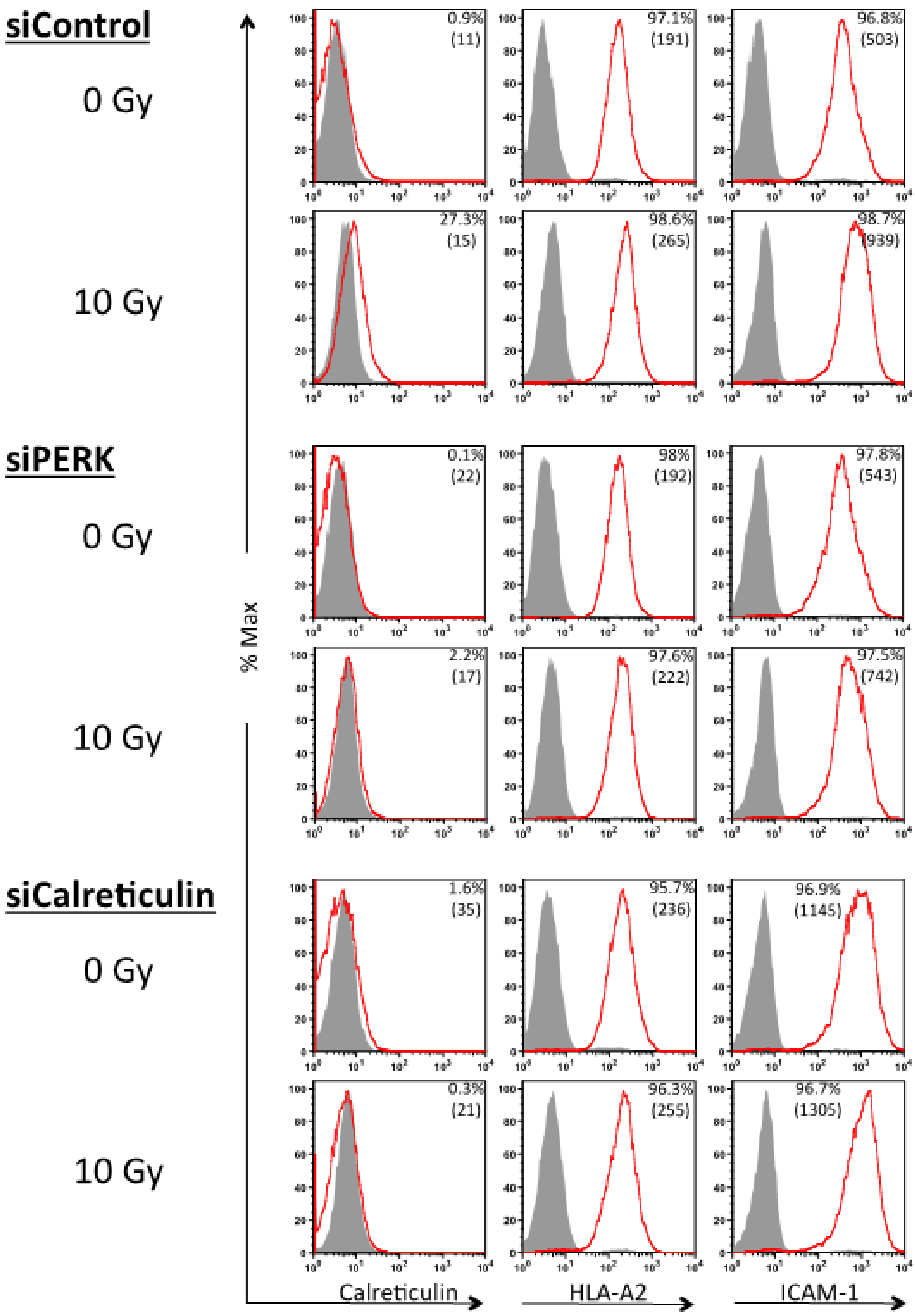

Supplemental Figure S1. Flow cytometry data supporting Figure 5. MDA-MB-231 carcinoma cells with control, calreticulin, or PERK siRNA knockdown were mock-irradiated (0 Gy; upper panels) or exposed to a single dose of 10 Gy (lower panels). After 48 h, tumor cell surface was examined by flow cytometry using antibodies specific for calreticulin, HLA-A2, and ICAM-1 (open histograms), or isotype-matched control antibodies (shaded histograms). Numbers indicate percentage of positive cells. Numbers in parentheses denote MFI. This experiment was repeated twice with similar results.

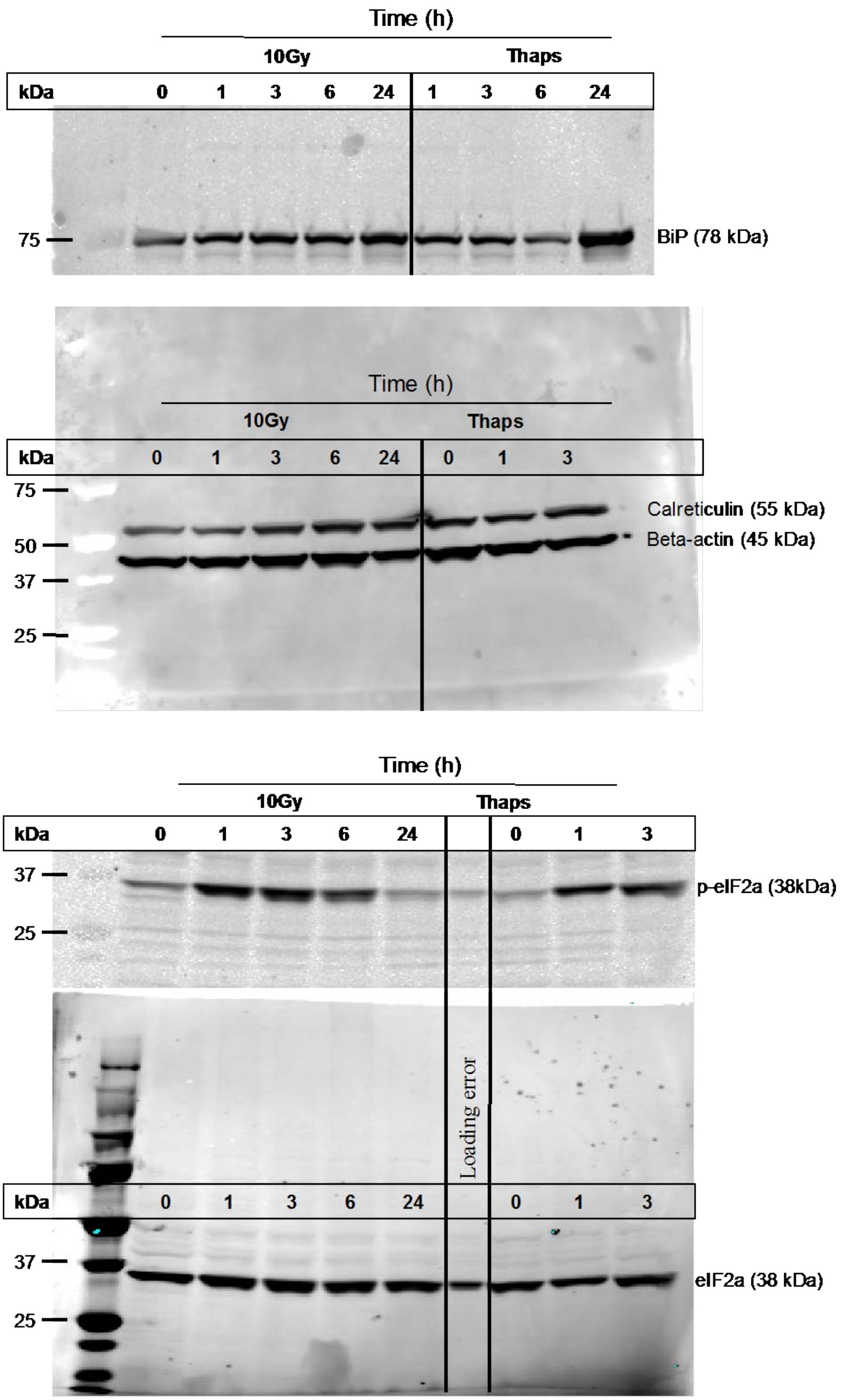

Supplemental Figure 2. Supporting western blots for Figure 6. At indicated times following exposure of MDA-MB-231 cells to 0 or 10 Gy, total cell lysates were prepared as described in **Materials and Methods**, and examined by western blotting to determine the expression of calreticulin, BiP, total eIF2a, and eIF2a-pSer51. Exposure to thapsigargin (THAPS, 0.5 uM/1h) was used as positive control for ER stress. Beta-actin was used as an internal control for total protein levels. Molecular weights of protein bands were determined relative to Odyssey molecular weight markers (kDa, left lane). This experiment was repeated twice with similar results.
