# Supplementary material for: Radiation-induced immunogenic modulation of tumor enhances antigen processing and calreticulin exposure, resulting in enhanced T-cell killing
Source: Oncotarget. 2013 Dec 31;5(2):403–16. doi: 10.18632/oncotarget.1719 (PMC3964216; doi:10.18632/oncotarget.1719)
Supplement: Supplementary file 2 [file oncotarget-05-403-s002.pdf]

Radiation-induced immunogenic modulation of tumor enhances antigen processing and calreticulin exposure, resulting in enhanced T-cell killing - Gameiro et al

			% Positive (MFI)	
			DMSO	THAPS
Antigen Processing Machinery	Intracellular	LMP2	94.5 (180)	95.5 (**303**)
		LMP7	75.8 (107)	81.2 (**156**)
		LMP10	88.6 (108)	93.4 (**191**)
		TAP1	91 (83)	92.6 (**117**)
		TAP2	95.7 (143)	96.7 (**223**)
		Calnexin	99 (260)	99.2 (**456**)
		Calreticulin	90.8 (78)	88.8 (**120**)
		Tapasin	92.6 (96)	90.6 (**168**)
	Cell-Surface	HLA-A2	98.8 (954)	97 (**1418**)
		ICAM-1	98.4 (1551)	95.4 (**2889**)
		CEA	9.4 (180)	11.8 (154)
		MUC-1	35.6 (51)	33.3 (**179**)

Supplemental Table 1. Tumor cells exposed to ER stress undergo immunogenic modulation. Human breast (MDA-MB-231) carcinoma cells were exposed to thapsigargin (0.2 uM/1h) or DMSO control. After 48 h, cells were analyzed by flow cytometry. Intracellular expression of indicated APM components and cell-surface expression of HLA-A2, ICAM-1, CEA, and MUC-1. Numbers indicate percentage of positive cells. Numbers in parentheses denote MFI. Bold denotes significant upregulation (≥ 30% increase in percent of cells or MFI not observed in isotype control vs. untreated cells).
